# Time-Series Interactions of Gene Expression, Vascular Growth and Hemodynamics during Early Embryonic Arterial Development

**DOI:** 10.1371/journal.pone.0161611

**Published:** 2016-08-23

**Authors:** Selda Goktas, Fazil E. Uslu, William J. Kowalski, Erhan Ermek, Bradley B. Keller, Kerem Pekkan

**Affiliations:** 1 Mechanical Engineering Department, Koc University, Istanbul, Turkey; 2 Kosair Charities Pediatric Heart Research Program, Cardiovascular Innovation Institute, University of Louisville, Louisville, KY, United States of America; Universita degli Studi di Bari Aldo Moro, ITALY

## Abstract

The role of hemodynamic forces within the embryo as biomechanical regulators for cardiovascular morphogenesis, growth, and remodeling is well supported through the experimental studies. Furthermore, clinical experience suggests that perturbed flow disrupts the normal vascular growth process as one etiology for congenital heart diseases (CHD) and for fetal adaptation to CHD. However, the relationships between hemodynamics, gene expression and embryonic vascular growth are poorly defined due to the lack of concurrent, sequential *in vivo* data. In this study, a long-term, time-lapse optical coherence tomography (OCT) imaging campaign was conducted to acquire simultaneous blood velocity, pulsatile micro-pressure and morphometric data for 3 consecutive early embryonic stages in the chick embryo. In conjunction with the *in vivo* growth and hemodynamics data, *in vitro* reverse transcription polymerase chain reaction (RT-PCR) analysis was performed to track changes in transcript expression relevant to histogenesis and remodeling of the embryonic arterial wall. Our non-invasive extended OCT imaging technique for the microstructural data showed continuous vessel growth. OCT data coupled with the PIV technique revealed significant but intermitted increases in wall shear stress (WSS) between first and second assigned stages and a noticeable decrease afterwards. Growth rate, however, did not vary significantly throughout the embryonic period. Among all the genes studied, only the MMP-2 and CASP-3 expression levels remained unchanged during the time course. Concurrent relationships were obtained among the transcriptional modulation of the genes, vascular growth and hemodynamics-related changes. Further studies are indicated to determine cause and effect relationships and reversibility between mechanical and molecular regulation of vasculogenesis.

## Introduction

Embryonic blood vessel development (vasculogenesis and angiogenesis) is regulated through continuous synthesis and remodeling processes. Inter- and intra-cellular signaling, under the influence of blood flow, regulates vascular microstructure and regenerative cascade of events, and thus the morphogenetic fate for the embryonic vessel network through genetic pathways [1]. The key regulatory processes are greatly influenced by changes in hemodynamic loading on the great arteries and veins, as well as by the growth factors that are important in arterial and venous differentiation [2, 3]. The genetic coding during embryonic vasculogenesis is controlled mostly by the endothelial cells, which respond to mechanical stimuli through various adaptive mechanisms depending on their vascular origin [4]. As a consequence, the arteries exhibit their unique identity during development compared to the veins [5].

In the early embryonic vasculature system, newly-formed endothelial cells are under the influence of blood shear stress and circumferential stress as physiological mechano-regulatory stimuli [6]. It is well known that cells react differently to these orthogonal forces [7]. Thus, the phenotypic behavior of these cells switch from one mechanism to another in a timely and flow-dependent manner while changing the biochemical nature, hence the growth path of the vessels. However, vessel growth and genetic mechanisms may not progress in perfect harmony with hemodynamic changes, leading to delays in response to flow-induced stress that are typically found within the embryonic vasculature [8]. Chick embryos have been frequently used in such studies as their cardiac morphogenesis resembles humans at the early stages and their manipulation is relatively easy. Although some of the hemodynamic parameters have been well assessed for the early chick embryonic cardiovascular system [9, 10], a correlative study relating the microvascular growth, genetic pathways and hemodynamics is still lacking in the literature.

One of the most useful modalities for embryonic cardiovascular biology studies is the optical coherence tomography (OCT), which enables non-invasive visualization of the vessels to 1–2 mm depth without the use of fluorescent markers, *in ovo* [11, 12]. Besides its superior capability in locating and quantifying the vascular structures, *in vivo* real-time OCT imaging technology has recently gained momentum in detecting the developmental malformations instead of routine and tedious pathological analyses [13]. When coupled with particle image velocimetry (PIV), OCT technique can yield the hemodynamics data for the normal and disturbed cardiac flows due to diseased state with high sensitivity [14].

Besides the 3D visual tracking of morphogenetic formations and deformations in early embryonic cardiovascular systems using OCT methodology, several attempts have been made to investigate the normal blood flow properties and their proximal relation to other morphogenetic parameters. In one of those studies, hemodynamics of fetal great arteries in mice was quantified through computational fluid dynamics (CFD) simulations, and it was seen the vessel size and flow-induced shear loading varied for different gestational periods [15]. It is also known that the altered hemodynamics within the microcirculation system causes changes in the genetic mechanisms in the developing embryos [16, 17].

Morphometric cues are dependent on intimate interplay among gene regulatory networks, flow-induced forces and the vascular growth mechanisms. Although it is difficult to draw direct implications between these factors, correlative scenarios may be of significant importance. Using chick embryo as model system, an OCT-based particle image velocimetry (PIV) system was used to obtain the hemodynamic parameters in the right vitelline arteries (RVAs). The flow- and growth-induced remodeling of the RVAs were investigated at three distinct embryonic stages, where rapid morphological changes were expected to occur. Here, we aim to unravel the complex interactive relations between the genetic response of the early embryonic cardiovascular cells and concurrent vessel growth under normal morphogenesis conditions.

## Materials and Methods

### Chick embryo incubation and right vitelline artery (RVA) collection

**Fig 1:**
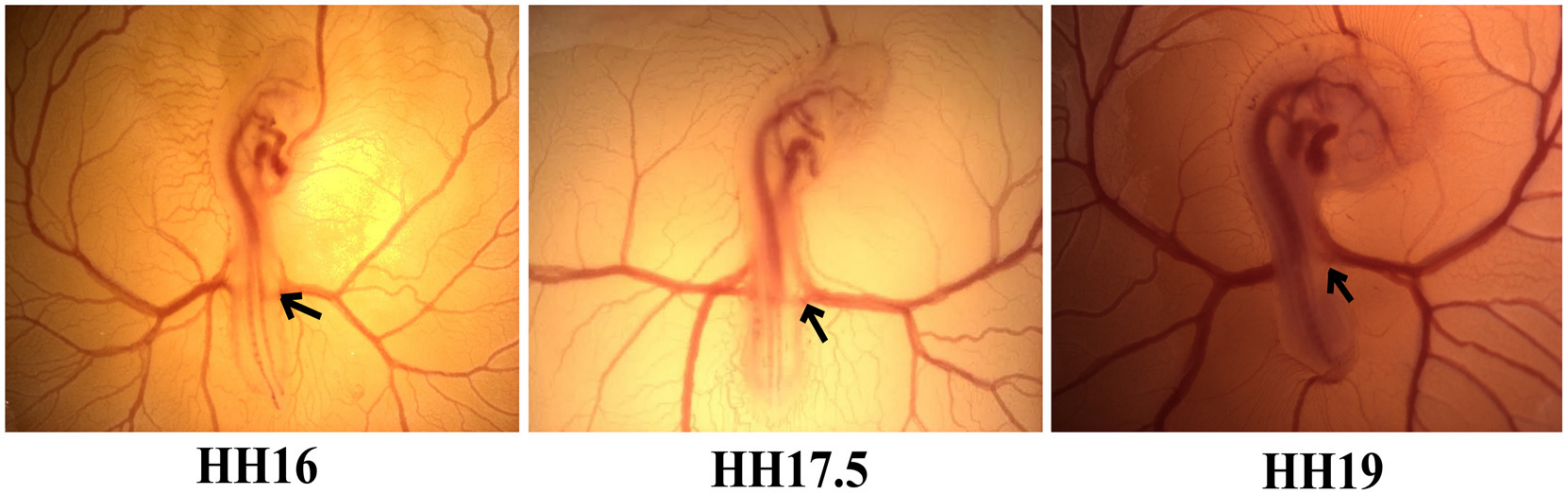
The chick embryo Hamburger and Hamilton (HH) stages investigated in this study. Arrows show the location of right vitelline artery (RVA) examined for the analyses.

Specific pathogen free (SPF) fertile White Leghorn eggs were incubated with the blunt side up at 37.5°C and 60–70% relative humidity (RH) to Hamburger and Hamilton (HH) stages HH16 (~2.3 days), HH17.5 and HH19 [18]. A small window was opened in the eggshell to gain access to the embryo. Right vitelline arteries (RVA) (as indicated in Fig 1) were dissected out with an in-house built dual micro-scissors and micro-tweezers (World Precision Instruments Inc.) and placed in cold Krebs solution containing (in mmol L^-1^): NaCl 110, KCl 5, NaHCO_3_ 24, KH_2_PO_4_ 1, MgSO_4_.7H_2_O 1, CaCl_2_ 2.5, glucose 10, and EDTA 0.02; pH 7.4. Tissues were then quickly transferred to RNAlater solution (Sigma-Aldrich) and kept at +4°C until analysis.

### RNA extraction and quantitative reverse transcription PCR (RT-qPCR)

The mRNA of pooled chick embryonic RVA samples (typically *n* = 12) from HH16, HH17.5 and HH19 was extracted using the GeneJET RNA purification kit (Thermo Scientific). cDNA was transcribed with Maxima First Strand cDNA Synthesis kit, and RT-qPCR reaction was performed on a PicoReal 96 Real-Time PCR system (Thermo Scientific). S1 Table summarizes the primer sequences used to amplify cDNAs encoding various proteins of four representative pathways that are important in maintaining vessel homeostasis during development: angiogenesis and apoptosis (VEGF-A, CASP-3, MMP-2 and TIMP-2), TGFβ/BMP signaling (BMP-2, TGFβ-3), mechano-sensitive (NOS-3, KLF-2 and ET-1), and cardiac developmental (VCAM-1, Nkx2-5). Glyceraldehyde-3-phosphate dehydrogenase (GAPDH) was used as the reference gene. All primer sequences were designed using the Beacon Designer software (version 8.13; Premier Biosoft International, Palo Alto, CA). The 2^-ΔΔC^_T_ method was constructed to compute the fold increase or decrease in mRNA expression levels between the stages [19].

### Optical coherence tomography imaging and morphometric analysis of embryos

An optical coherence tomography (OCT) system (Thorlabs Spectral Domain Ganymede, Thorlabs, Inc., NJ), enclosed in a custom-built environmental chamber maintained at 37°C and 60% humidity, was used for imaging and 3D reconstruction of the vascular RVA lumen *in ovo* as previously described [20]. During the time-lapsed experiments, embryos were continuously imaged through a 12 bit high-sensitivity CCD camera attached to the system.

We acquired 2D single-time-point OCT images at HH16, 17.5, and 19 for additional radius measurements at these three stages. A long-axis view at the center of the vessel was used and we took 10 radius measurements per RVA. A total of *n* = 15 embryos were measured per stage. Measurements were normalized to the mean HH16 value for analysis.

### Assessment of hemodynamic parameters

#### Velocity profiles

Images obtained by time-resolved OCT-particle image velocimetry (PIV) technique were used to evaluate velocity profiles within the right vitelline arteries at stages HH16, HH17.5 and HH19 [14, 21]. Velocity data was extracted with DaVis 8.1.0 software (LaVision, Inc., Germany). Single frames taken at the center of the vessels were acquired with OCT technique and time series of single frames PIV algorithm was used as the vector calculation method. The vessel region was manually traced and a mask was applied to constrain vector calculation. Smoothing was applied to improve the vectors. Poor neighboring vectors were removed if greater than the 2 root mean square (RMS) magnitude and multi-pass post-processing median filtering was reinserted for vectors less than 3 RMS of neighboring vectors [14]. A multi-pass interrogation window was used with decreasing size. The size of the first interrogation window was 128x128 pixels with 50% overlap (1 pass), and the size of the second interrogation window was 48x48 pixels with 75% overlap (2 passes). During the vector post processing, median filter was applied.

#### Wall shear stress calculation

Blood flow data were collected at different frame speeds for each embryo: 200 frames at 67 frames per second (fps), 200 frames at 55 fps, and 100 frames at 33 fps including 6–7 cardiac cycles. Data sets with 67 fps were used for hemodynamics calculations. Each cardiac cycle included approximately 25 time points. Mean velocities of the time points were acquired by averaging the velocity vector distribution. Phase-locked averaging was performed over each time series to obtain a single cardiac cycle per embryo. The same procedure was applied for the 55 fps and 33 fps data to validate results obtained from the cardiac cycle at 67 fps.

Mean peak wall shear stress (WSS) for each stage was calculated from phase-locked averages of cardiac cycles at each stage (*n* = 5). The flow in the vessels was laminar and possessed Poiseuille flow profile in each frame of the data used for analyses. Mean peak velocities of phase-locked averages of cardiac cycle for each stage were used as V_mean_ for the calculation of the peak WSS values. Dynamic viscosity values used during peak WSS calculations were extracted from literature [22]. Fluctuations in the cardiac cycles were investigated via calculating percentage backflow and Oscillatory Shear Index (OSI).

#### Vitelline artery blood pressure data

A dual-channel 900A micropressure measurement system (World Precision Instruments, Sarasota, FL) was used to monitor the pulsatile blood pressure in the RVAs. Pressure data was collected immediately after opening the shell to minimize the effects of environmental hypothermia. A 2–5 μm glass capillary was inserted into the artery underneath the vitelline vein. Lab-Trax hardware and LabScribe software were used for the measurements with system sensitivity set at 0.1 mmHg. The system was calibrated each time before the new measurement in the calibration chamber and an offset value was added to measurement values. The micro-pressure system was also validated with DPM2 plus pressure meter (Fluke Biomedical, Everett, WA) under static pressures integrated with a glass micro-manometer.

### Statistical analysis

Statistical analyses were performed with IBM SPSS Statistics v24. One-way univariate ANOVA (and the accompanying F-test) was employed to analyze the differences in growth, hemodynamic parameters (shear stress and pressure) and gene expression levels between the embryonic stages. When the omnibus ANOVA F-test was significant, pairwise t-tests were applied as post hoc tests, and Bonferroni adjustment was performed when necessary. Statistical significance level was set to 0.05 for all tests (i.e., a p-value is deemed significant if it is less than 0.05). All summary values were presented as mean ± standard deviation.

## Results

### Vitelline artery growth rates

**Fig 2:**
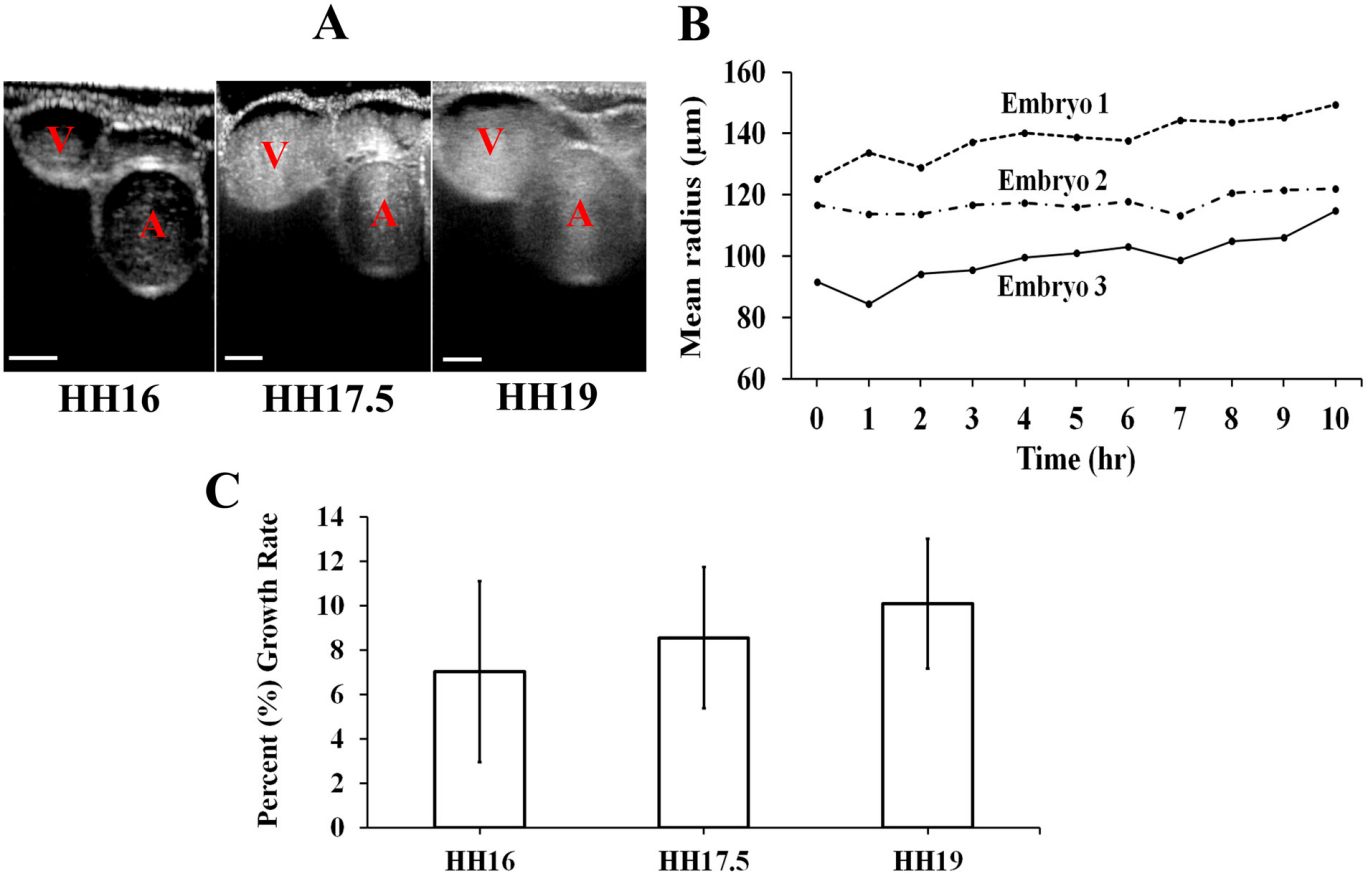
**(A)** Radial projections of the RVA vessels showing the vitelline artery (A) and the vitelline vein (V) at stages HH16, HH17.5 and HH19. Scale bars represent 100 μm. **(B)** Continuous growth data for the embryos during the 10 hr-period (*n* = 3), **(C)** Percent (%) growth rate of mean radius for the RVAs (*n* = 5 for each stage).

The radial growth of the chick embryonic RVAs was quantified using the OCT-PIV imaging methodology. The resolution and sensitivity of our OCT system (Telesto 2 Spectral Domain OCT, Thorlabs Inc.) was sufficient to reliably identify the boundaries of the RVA lumen (Fig 2A). Continuous hourly mean vessel radius (average of minor and major radii) recordings over the 10 hr time course depicted a gradual increase with time (*n* = 3) (Fig 2B). The instantaneous percent (%) vascular growth rate, on the other hand, remained constant throughout the time course (*p* = 0.099) (Fig 2C). Although our time-lapse data shows a general increasing trend in RVA radius, comparisons between normalized radii at HH16, 17.5, and 19 did not reveal any significant differences (*p* > 0.05, data not shown). This result indicates that the growth rate is too gradual to produce a statistical change over this 10-hour window.

### Mechanical loading in the RVA

**Fig 3:**
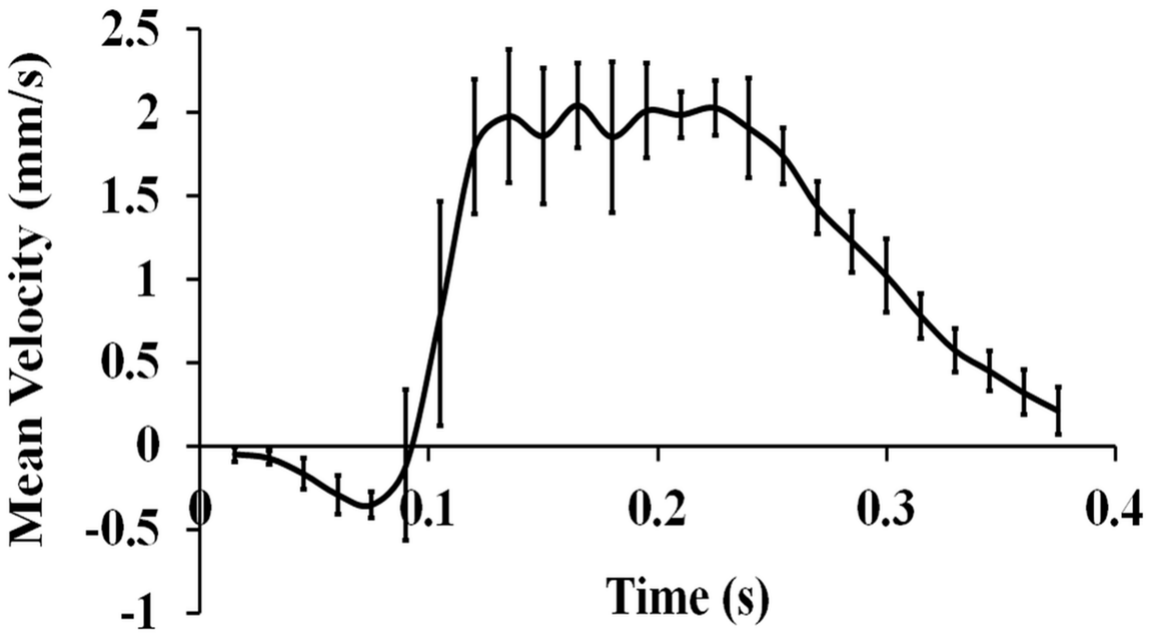
Representative phase-locked average of a cardiac cycle at stage HH17.5 (*n* = 5). Cardiac cycles are composed by average velocity values of velocity field at 25 time points whose standard deviations are depicted with error bars.

The supplementary file in S1 Movie demonstrates the pulsatile nature of the blood flow within the chick embryonic RVAs. The pulsatile phase-locked cardiac cycle at HH17.5 is shown in Fig 3, where the time points are the average of velocity magnitudes of parabolic flow profile and variations in mean velocities are presented at each phase-locked time point in the cardiac cycle. Negative back flow was observed in all embryos (12%, 3.64%, and 5.29% backflow for HH16, HH17.5, and HH19, respectively). It was seen that the mean velocity values mostly deviated when forward pumping of the embryonic heart has started. The peak velocity region of forward flow (positive velocity field of cardiac cycle) remained at around 2 mm/s for approximately 0.12 seconds.

**Fig 4:**
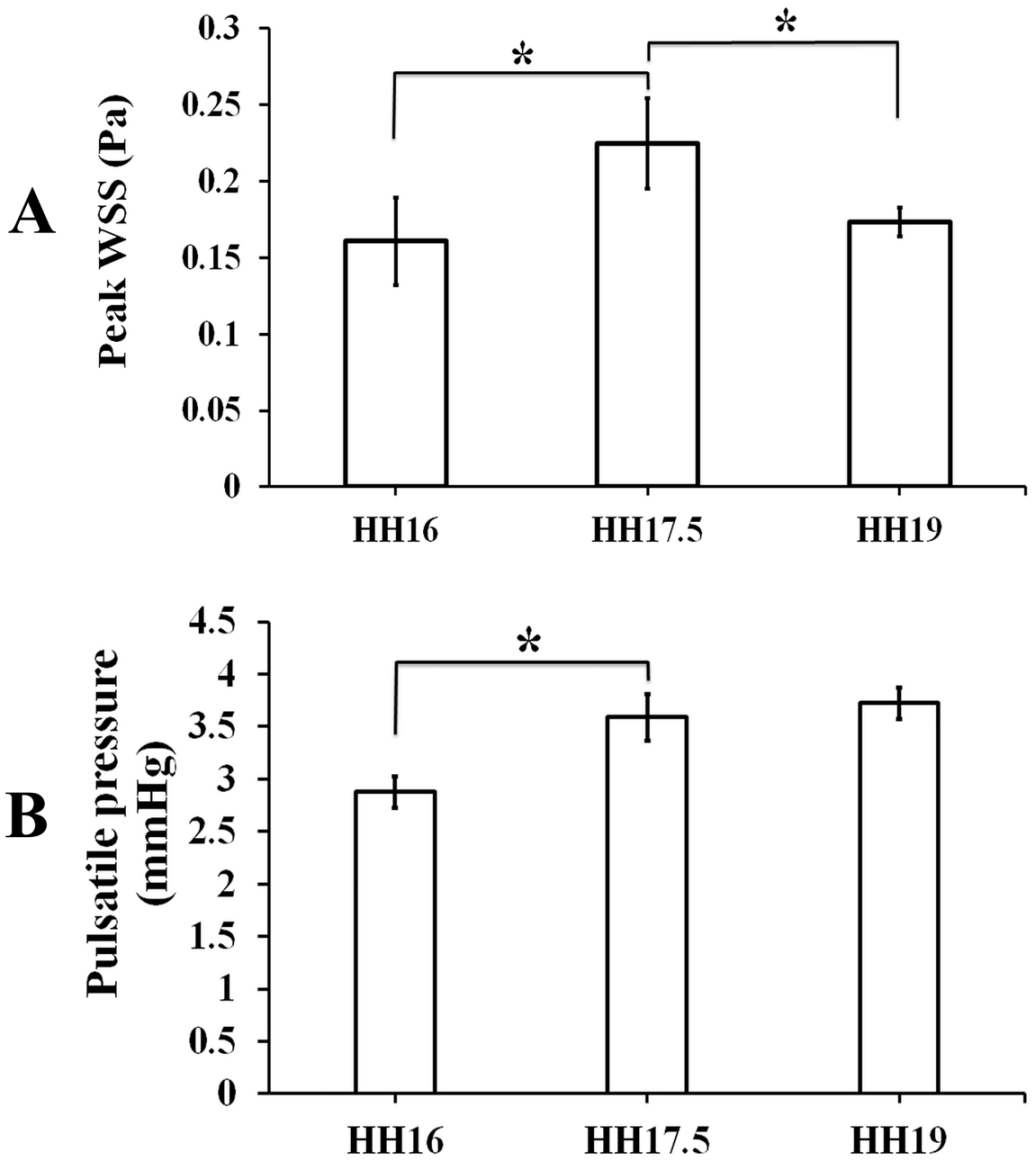
**(A)** Wall shear stress (WSS) values reached within the right vitelline arteries at stages HH16, HH17.5 and HH19 (*n* = 5). **(B)** The mean pulsatile blood pressure values measured for the right vitelline artery vessels at stages HH16, HH17.5 and HH19. Solid circumferential stress is known to be directly proportional with pressure values. The mean pressure, thus the circumferential stress values, increased significantly towards HH17.5, then remained insignificantly changed when HH19 was reached (*n* = 4). * denotes statistically significant differences (*p* < 0.05).

The cardiac period at HH16 was 0.46 ± 0.01 s, which then decreased significantly towards HH17.5 (0.39 ± 0.02 s), and remained almost unchanged afterwards. There were no significant changes in OSI between the stages (data not shown for brevity). The peak WSS increased significantly from HH16 to HH17.5 (*p* = 0.0004), and decreased significantly from HH17.5 to HH19 (*p* = 0.007) (Fig 4A). Likewise, the mean right vitelline arterial pressures for stages HH16, HH17.5 and HH19 were recorded as 2.87 ± 0.15 mmHg, 3.59 ± 0.22 mmHg, and 3.72 ± 0.15 mmHg, respectively (*n* = 4). The pressure increase from embryonic stage HH16 to HH17.5 was significant (*p* = 0.013) (Fig 4B).

### Gene expression

#### Angiogenesis- and Apoptosis-related gene expression

**Fig 5:**
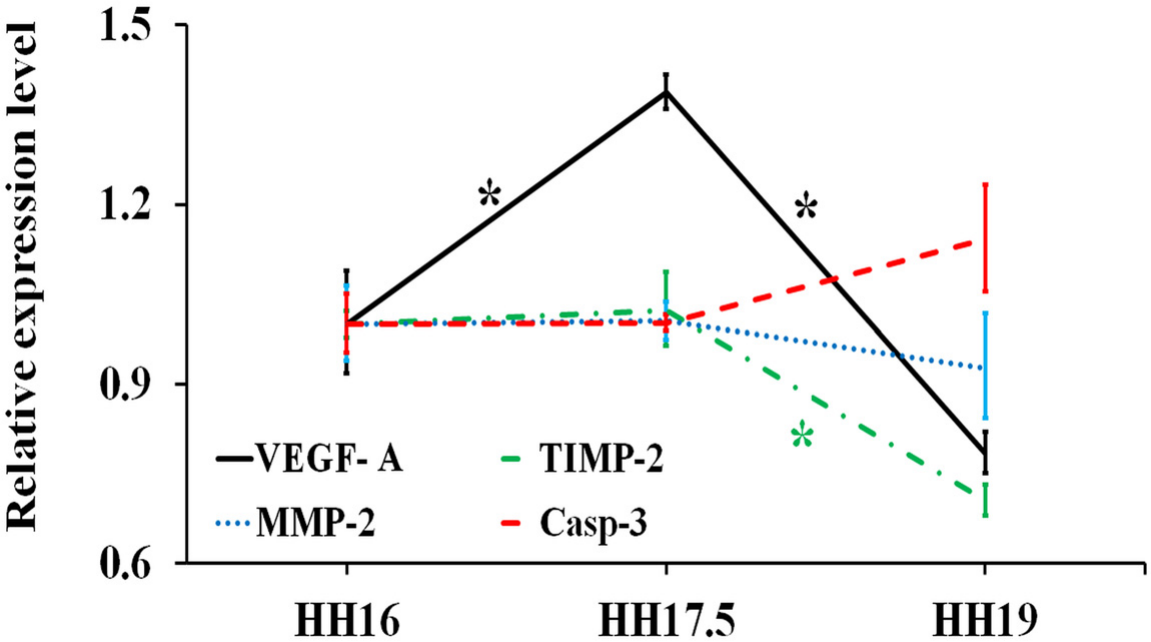
mRNA levels for angiogenesis- and apoptosis-related genes (VEGF-A, TIMP-2, MMP-2, CASP-3) at stages HH17.5 and HH19 relative to HH16 (*n* ≥ 4). Fold changes were obtained by normalizing to the housekeeping gene GAPDH. * indicates statistically significant difference (*p* < 0.05).

The expression patterns for VEGF-A, MMP-2, TIMP-2 and Casp-3 were studied over the 10 hr period (Fig 5). There was a 1.39-fold increase in VEGF-A expression for HH17.5 RVA compared to HH16 (*p* = 0.007), then a significant decrease in expression level towards HH19 (*p* = 2.34E-5). MMP-2 expression however remained similar between stages HH17.5 and HH19. For TIMP-2 expression level, a significant change was observed between HH17.5 and HH19 (*p* = 0.004). On the other hand, Casp-3 gene activation was insignificantly influenced by tissue growth.

#### Cardiac developmental pathway

**Fig 6:**
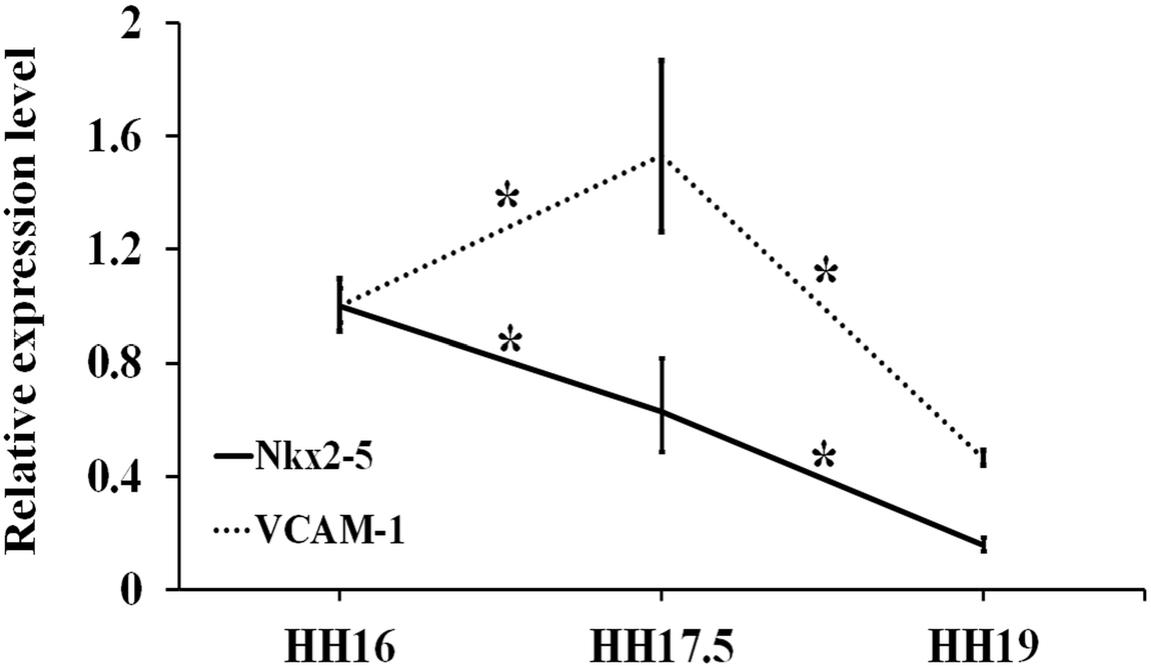
Expression levels for cardiac developmental genes (Nkx2-5, VCAM-1) at stages HH17.5 and HH19 relative to HH16 (*n* ≥ 4). Fold changes were obtained by normalizing to the housekeeping gene GAPDH. * indicates statistically significant difference (*p* < 0.05).

Expression levels for the cardiac development-related genes Nkx2-5 and VCAM-1 for the three embryonic stages were investigated (Fig 6). VCAM-1 expression showed a significant increase towards HH17.5 (*p* = 0.04), and a subsequent decrease towards HH19 (*p* = 0.02). Nkx2-5 mRNA expression demonstrated a continuous decline from HH16 to HH17.5 (*p* = 0.03), and from HH17.5 to HH19 (*p* = 5.82E-4).

#### Mechano-sensitive gene expression

**Fig 7:**
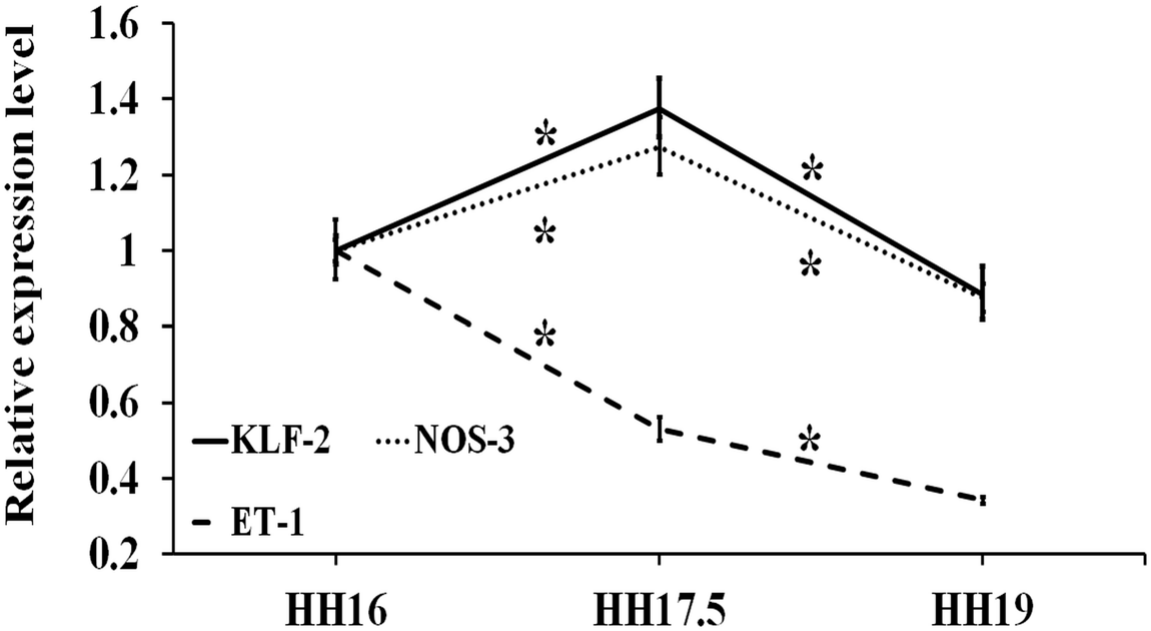
Expression profiles for mechano-responsive genes (KLF-2, NOS-3, ET-1) at stages HH17.5 and HH19 relative to HH16 (*n* ≥ 4). Fold changes were obtained by normalizing to the housekeeping gene GAPDH. * indicates statistically significant difference (*p* < 0.05).

In regard to the mechano-responsive genes, expression levels for KLF-2, NOS-3, ET-1 were quantified (Fig 7). A similar expression trend was observed for KLF-2 and NOS-3: an increase towards HH17.5 (KLF-2: *p* = 0.004; NOS-3: *p* = 0.009) and decrease towards HH19 (KLF-2: *p* = 6.15E-5; NOS-3: *p* = 0.003). The ET-1 expression decreased from HH16 to HH17.5 (*p* = 0.003), then further decreased to HH19 (*p* = 0.005).

#### TGFβ/BMP signaling pathway-related gene expression

**Fig 8:**
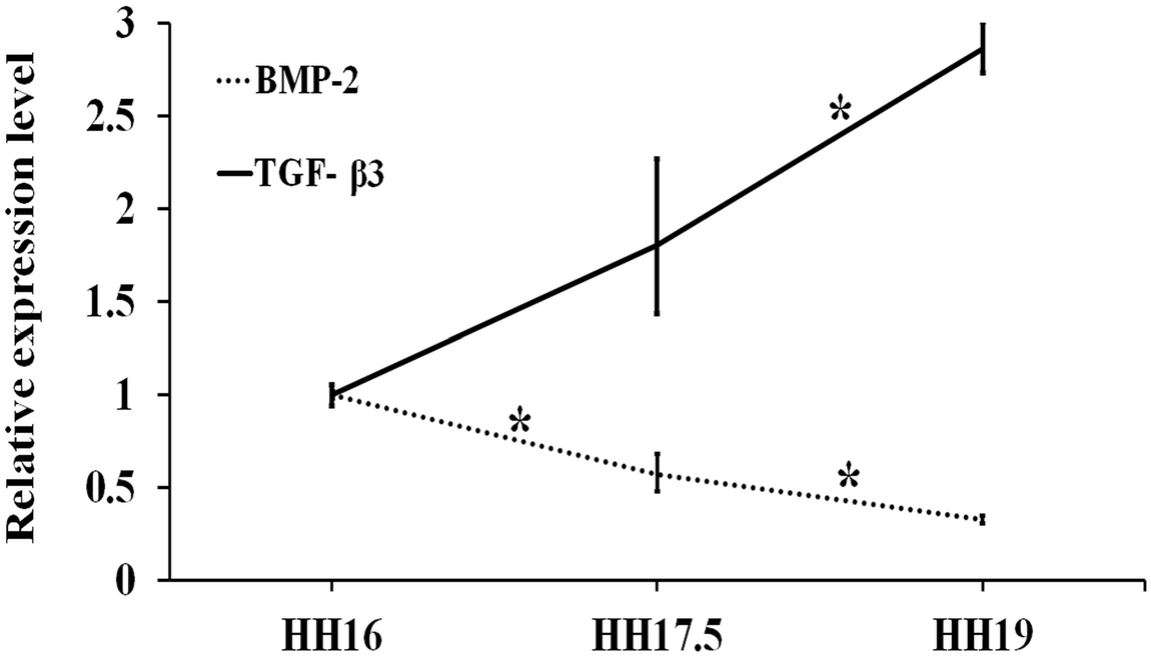
mRNA levels for TGF-^®^/BMP signaling genes (BMP-2, TGF-β3) at stages HH17.5 and HH19 relative to HH16 (*n* ≥ 4). Fold changes were obtained by normalizing to the housekeeping gene GAPDH. * indicates statistically significant difference (*p* < 0.05).

The BMP-2 mRNA extracted from chick embryonic RVAs showed a tendency to continuously decrease from HH16 to HH17.5 (*p* = 0.01) and further to HH19 (*p* = 0.03). The TGF-β3 expression, on the other hand, changed significantly (increased) only between the stages HH17.5 and HH19 (*p* = 0.02) (Fig 8).

A summary of the expression trends for the genes studied is tabulated in Table 1.

**Table 1:** Changes in the genetic activity of the extracted right vitelline arteries between the Hamburger and Hamilton (HH) stages.

	HH16-17.5	HH17.5–19
**Angiogenesis and Apoptosis**		
VEGF-A	⬆	⬇
MMP-2	No change (NC)	NC
TIMP-2	NC	⬇
CASP-3	NC	NC
**Cardiac development**		
Nkx2-5	⬇	⬇
VCAM-1	⬆	⬇
**Mechano-sensitive**		
KLF-2	⬆	⬇
NOS-3	⬆	⬇
ET-1	⬇	⬇
**TGFβ/BMP signaling**		
BMP-2	⬇	⬇
TGF-β3	NC	⬆

*VEGF-A*, vascular endothelial growth factor-α; *CASP-3*, caspase-3; *BMP-2*, bone morphogenetic protein-2; *TGFβ-3*, transforming growth factor β-3; *VCAM-1*, vascular cellular adhesion molecule-1; *NOS-3*, nitric oxide synthase-3; *KLF-2*, krüppel-like factor-2; *ET-1*, endothelin-1; *MMP-2*, matrix metalloproteinase-2; *TIMP-2*, tissue inhibitor of metalloproteinase-2; *Nkx2-5*, cardiac homeobox protein; *GAPDH*, Glyceraldehyde 3-phosphate dehydrogenase. Up (⬆) and down (⬇) arrows indicate up-regulation and down-regulation, respectively.

## Discussion

The present multi-modal study on embryonic arterial development demonstrated that the early vitelline arteries possess high potential to rapidly remodel structurally and functionally. The selected embryonic window features two distinct mechanical loading levels, which can be correlated with the corresponding pathway trends. At stage HH17.5, the embryonic heart loop has gained its s-shape where the more prominent pumping action of the heart and the active contractions of the outflow tract (OFT) caused higher blood velocities in the vitelline network, hence increased WSS. On the other hand, at stage HH19, the blood flow rate has decreased as more blood accumulation started to occur in the atrium thus decreasing the WSS [23]. In accordance with the *in vivo* findings of Groenendijk *et al*., we found a direct proportionality between the synthesis rates of NOS-3 and KLF-2, and the applied shear stress on the vessel walls [24]. Similar to their observations, the vasoconstrictor ET-1 gene expression indicated an inverse relationship with shear and was down regulated by increased WSS from HH16 to HH17.5. Our results showed KLF-2 and NOS-3 responded relatively fast and reversibly to changes in shear stress during the time course. The on-going down regulation of ET-1 between HH17.5–19 in a lesser extent might have possibly occurred as a result of a time and response lag to shear within this period. The delay in response of ET-1 might be due to its strong dependence on the profound effect of the phase lag between circumferential stress (which strongly correlates with pulsatile pressure) and shear stress [25]. Shear and pressure load within the chick embryonic RVAs increased synchronously until HH17.5, then a delay between shear and pressure became obvious between stages HH17.5 and HH19 where the extent of change in shear stress values was greater compared to pressure. Therefore, ET-1 synthesis may be pressure-sensitive, rather than WSS, unlike KLF-2 and NOS-3. Furthermore, the induction of NOS-3 expression and suppression of ET-1 expression at elevated KLF-2 expression levels may be either due to the direct influence of KLF-2 gene or the steady laminar stress in the arterial vessels as described earlier [26, 27]. Finally, the up-regulation or down-regulation of KLF-2 might just have been controlled as a consequence of the developmental stages.

On the other hand, the *in vivo* expression of VEGF-A followed both shear stress and pulsatile pressure trends between stages HH16 and HH17.5. As with the increased shear and pressure levels, the endothelial cells residing along the arterial endothelium undergo a switch-on mechanism to activate the synthesis of more of this growth factor for the continuation of their survival [28]. Beyond stage 17.5, the direct influence of shear on the endothelial release of this growth factor persisted and the pressure effect ceased. In the chick RVAs, the consequent induction and inhibition of VCAM-1 expression during the embryonic stages investigated in this study may as well be correlated with VEGF-A synthesis rate, as VEGF-A is known to promote the production of VCAM-1 protein by endothelial cells [29]. KLF-2 is also known as an anti-inflammatory gene that down regulates endothelial synthesis rates for the proinflammatory agent VCAM-1, but the KLF-2 independent expression of VCAM-1 indicated KLF-2 mediated its anti-inflammatory action through control of some other molecule [30].

Nkx2-5 plays a pivotal role in cardiac mesoderm expansion and the development of cardiovascular structures, and is one of the earliest transcription factors expressed during morphogenetic processes. The transcript level for Nkx2-5 was initially higher at stage HH16 and down regulation of this gene was observed for later stages in the chick RVAs. Notably, it is known that BMP signaling is critical for the synthesis of Nkx2-5 protein during cardiogenesis [31]. The steady decreasing trend in the BMP-2 synthesis rates in our study might have thus affected the expression levels of Nkx2-5 with a higher rate of decrease. On the contrary, the expression levels for TGF-β3, an important member of the TGF-β/BMP signaling cascade in the chick RVAs, increased continuously through the time course. An earlier study on the chick embryo also indicated the secretion of TGF-β3 mRNA by the endothelial cells of vitelline arteries at stage HH15-20, but TGF-β3 synthesis trend with developmental age was missing in that study [32].

In our study, we did not observe a significant variation in the expression levels for the essential extracellular matrix (ECM) protease MMP-2 activity that plays a key role in the vitelline artery morphogenesis. In a previous study with quail model, the change in MMP-2 mRNA synthesis was more noticeable during the early stages (stage 12) at the ventricle, but similar MMP-2 synthesis rates were reached for the later stages [33]. Also, one may elucidate the almost constant change in the synthesis rates of MMP-2 and its inhibitor enzyme TIMP-2 in the chick RVAs between stages HH16 and HH17.5 by concluding these genes were more responsive to vascular growth rate rather than hemodynamic loading. After HH17.5, the concomitant decrease in the MMP-2 expression levels with TIMP-2 might be as a consequence of the critical cellular processes to maintain vascular homeostasis. Additionally, the change in TIMP-2 and MMP-2 expression levels may become more pronounced for prolonged hemodynamic loading on the vessels at later stages. The up- or down-regulation of these ECM proteases could be observed by the end of a 7-day *ex vivo* perfusion of human saphenous veins [34].

Various approaches to quantify the flow parameters in the chick embryo have made it a valuable model to explore vasculogenesis and angiogenesis relevant to humans [35–37]. While the majority of our results are consistent with previous studies, a few proved to be contradictory. It was reported earlier that the steady laminar flow was not achieved in the early chick embryo before stage HH12 and the flow demonstrated backflow and mixing until then [38]. However, the flow measurements extracted from our PIV processing methodology showed 12% backflow for stage HH16, then less backflow in blood stream at stage HH17.5, and further less for stage HH19. This was indicative of the continuity of fluctuations and unsteady flow profiles in blood flow until later stages than HH12.

In ultra thin-walled vessels like early embryonic arteries and veins, pulsatile blood pressure is directly proportional to the circumferential stress that plays a vital role in vascular remodeling. Few studies have been conducted in the chick embryo to quantify the vitelline blood pressure that causes the vessel to contract and expand throughout the cycle. Hu and Clark reported the mean vitelline arterial pressures for stages HH16, HH18 and HH21 as 0.49±0.03 mmHg, 0.71±0.02 mmHg, and 0.90±0.02 mmHg, respectively, and observed the systolic and diastolic pressures increased linearly with incubation time [39]. Consistent with their data, we obtained similar hemodynamic pressure waveforms from the RVAs. However, our pressure values demonstrated a slight upward shift. This variation might have resulted as a consequence of different models of micro-pressure measurement systems used and calibration schemes adapted for these challenging systems.

The individual time-lapsed hourly growth curves consistently show a general increasing trend and the rate of change in vessel radius of the RVAs remained nearly constant throughout the growth period. However, a significant difference in RVA radius was not observed from 2D measurements of a larger sample size possibly due to the accuracy of OCT. Note that the steady growth rate, is a “slope calculation” based on “multiple” time-points whereas the diameter measurements are obtained for one single instant during development. Furthermore, the increase in blood velocity and shear stress values along the vessel towards stage HH17.5, and the subsequent decrease may not be at critical levels to generate a growth response [40]. In addition, at these early embryonic stages, there may be an intrinsic stimulus for vascular growth, mitigating any biomechanical response to changes in shear stress.

### Technical Limitations

The OCT depth of field is dependent on the attenuation of light through the sample. For *in ovo* imaging of the chick embryo, the large reflection at the air-albumin interface and the red blood cells within vessels result in an imaging depth of 1–1.5 mm. In this study, the location of the vitelline artery at stage HH19 was typically 1–2 mm below the surface of the albumin, which limited our acquisition of imaging data for PIV and 3D reconstructions at this time point. In most embryos, the vitelline artery was located directly underneath the vitelline vein, which further obstructed imaging due to the red blood cells.

At the investigated stages, the vessel walls were approximately 1–2 cell layers thick [41]. In early embryonic wild-type zebrafish, it was seen that while taking the wall motion into account, the change in the first aortic arch (AA1) diameter due to compliance was about 10% and lied within the error range of diameter calculations [14]. Hence, discounting wall motion would not reduce the PIV accuracy. Therefore, we did not include a measure of mural thickness and ignored wall motion in our analysis. Additionally, since the displacement of the vitelline artery wall during pulsation is less than or at the limit of the OCT resolution of 4 μm, any error introduced by this assumption would be small. Furthermore, we are not currently aware of any PIV studies of chick embryo vitelline vessels that include wall motion. While we used anatomical landmarks and placed sutures at the imaging site, the use of fiducial markers may improve the accuracy of the scanning location when performing long-term imaging [42].

The present study highlighted that the gene expression levels may not be synchronized in time. Particularly for the genes that are downstream of the pathway, the WSS increase can lead to changes in expression levels at different embryonic times. Thus, a statistical correlative analysis regarding the possible interactions between gene expression and pressure/shear has not been attempted, as this kind of analysis requires finely resolved embryonic time points.

One other limitation for the PIV measurement might be the assumption that the blood flow streamlines in the early embryonic RVAs were straight (rather than curved). Still, the PIV data extracted from the RVAs demonstrated mostly straight flow characteristics. As a consequence, that assumption did not bring any error to our calculations.

## Supporting Information

S1 Movie Blood stream within the right vitelline arteries. Movie shows a projected view of the pulsatile back-and-forth flow of blood cells that are represented as blue dots at a time point in the vessel.
(MP4)

S1 Table Forward (F) and reverse (R) primer sequences for quantitative reverse transcriptase polymerase chain reaction (RT-qPCR). (DOCX)
