# Supplementary material for: Time-Series Interactions of Gene Expression, Vascular Growth and Hemodynamics during Early Embryonic Arterial Development
Source: PLoS One. 2016 Aug 23;11(8):e0161611. doi: 10.1371/journal.pone.0161611 (PMC4994943; doi:10.1371/journal.pone.0161611)
Supplement: S1 Table — (DOCX) [file pone.0161611.s002.docx]

**S1 Table. Forward (F) and reverse (R) primer sequences for quantitative reverse transcriptase polymerase chain reaction (RT-qPCR).**

| **Gene** | **Primer sequence (5’-3’)** | **T_m_ (°C)** |
| --- | --- | --- |
| **VEGF-α** | F: CTTTCTGCTCACTTGGATCCAC | 60.3 |
|  | R: GTCTCAATTGTCCTGCAGAAGC | 60.3 |
| **CASP-3** | F: CAGATGCAAGATCTTTCCCTGG | 60.3 |
|  | R: CCGGTATCTCGGTGGAAGTTC | 61.8 |
| **BMP-2** | F: TGTGGTGGAGGTGGTTCA | 56.0 |
|  | R: GGTGTCCCTTGCCATCATG | 58.8 |
| **TGFβ-3** | F: TACCTCAGTGGCAGGAATGT | 57.3 |
|  | R: GTATGGCAAGGGCAGTGTATG | 59.8 |
| **VCAM-1** | F: TCATGCCCATTTCACTGACTCG | 60.3 |
|  | R: GATAATCTGAACAGCTGGGACACC | 62.7 |
| **NOS-3** | F: TGGTACATGAGCAGCGAGAT | 57.3 |
|  | R: TCCAGAGGGACGAAGTCTTAC | 59.8 |
| **KLF-2** | F: CCCACGCAAAGAGGATGAAG | 59.4 |
|  | R: GTTCGGGTTTGTCTGGTAGAAG | 60.3 |
| **ET-1** | F: TGTTCCCTATGGTCTTGGAGGC | 56.8 |
|  | R: AGGTTTTCTCTGCTGTGGACTGAG | 55.7 |
| **MMP-2** | F: GTCACGCCACTGAGATTTAACC | 60.3 |
|  | R: CTCTAACCACTTGCCCTTCTCC | 62.1 |
| **TIMP-2** | F: CCATCAAGCGAATCCAGTACG | 59.8 |
|  | R: ACTCCTTCTTACCTCCTGTGTC | 60.3 |
| **Nkx2-5** | F: CTGTGACGACGACTCCCTT | 58.8 |
|  | R: GAACGCCTCCTGCTTGAAG | 58.8 |
| **GAPDH** | F: GATTCTACACACGGACACTTCA | 58.4 |
|  | R: CTGAGGGAGCTGAGATGATAAC | 60.3 |

*VEGF-α*, vascular endothelial growth factor-α; *CASP-3*, caspase-3; *BMP-2*, bone morphogenetic protein-2; *TGFβ-3*, transforming growth factor β-3; *VCAM-1*, vascular cellular adhesion molecule-1; *NOS-3*, nitric oxide synthase-3; *KLF-2*, krüppel-like factor-2; *ET-1*, endothelin-1; *MMP-2*, matrix metalloproteinase-2; *TIMP-2*, tissue inhibitor of metalloproteinase-2; *Nkx2-5*, cardiac homeobox protein; *GAPDH*, Glyceraldehyde 3-phosphate dehydrogenase; *T_m_*, melting temperature.
